# Tracing the locality of prisoners and workers at the Mausoleum of Qin Shi Huang: First Emperor of China (259-210 BC)

**DOI:** 10.1038/srep26731

**Published:** 2016-06-02

**Authors:** Ying Ma, Benjamin T. Fuller, Weigang Sun, Songmei Hu, Liang Chen, Yaowu Hu, Michael P. Richards

**Affiliations:** 1 Department of Human Evolution, Max Planck Institute for Evolutionary Anthropology, 6 Deutcher Platz, D-04103 Leipzig, Germany; 2 Department of Archaeology and Anthropology, University of Chinese Academy of Sciences, Beijing 100049, China; 3 Shaanxi Provincial Institute of Culture Relics and Archaeology, Xi’an, Shaanxi 710054, P. R. China; 4 Institute of Archaeology, Northwest University, Xi’an, Shaanxi 710069, P. R. China; 5 Key Laboratory of Vertebrate Evolution and Human Origins of Chinese Academy of Sciences, Institute of Vertebrate Palaeontology and Palaeoanthropology, Chinese Academy of Sciences, Beijing 100044, China; 6 Department of Archaeology, Simon Fraser University, Burnaby, BC V5W 1S6, Canada

## Abstract

The mausoleum complex of the First Emperor of China, Qin Shi Huang (259-210 BC), is one of the most famous and important archaeological sites in China, yet questions remain as to how it was constructed and by whom. Here we present isotopic results of individuals from the Liyi (n = 146) and Shanren sites (n = 14), both associated with the mausoleum complex. Those buried at Liyi represent the local workers/inhabitants of the Qin population, and the δ^13^C (−8.7 ± 1.5%) and δ^15^N (10.3 ± 0.7%) values indicate that they consumed predominately millet and/or domestic animals fed millet. In contrast, the Shanren individuals were prisoners forced to construct the mausoleum (found buried haphazardly in a mass grave and some in iron leg shackles), and their δ^13^C (−15.4 ± 2.9%) and δ^15^N (8.0 ± 0.6%) results indicate a more mixed C_3_/C_4_ diet, with possibly less domestic animals and more wild game protein consumed. This pattern of decreased millet consumption is also characteristic of archaeological sites from southern China, and possible evidence the Shanren prisoners originated from this region (possibly the ancient Chu state located in modern day Hubei Province and parts of Hunan and Anhui Provinces). Further, this finding is in agreement with historical sources and is supported by previous ancient DNA evidence that the mausoleum workers had diverse origins, with many genetically related to southern Chinese groups.

As the First Emperor of China, Qin Shi Huang (259-210 BC), brought to conclusion the tumultuous Warring States Period (475-221 BC) and successfully established the first unified Empire of China: the Qin Dynasty^1^,^2^. The magnificent mausoleum complex of Qin Shi Huang (including the life sized Terracotta Warrior Army) is considered one of the most important archaeological sites of China, and has been described as the 8^th^ of wonder of the world^3^. Chinese historical records indicate that as soon as Qin Shi Huang gained control of the Qin State (246 BC), he started building the mausoleum at the northern portion of Lishan Mountain^4^, near the present day city of Xi’an (Fig. 1). Massive amounts of planning, labor and capital were diverted to the construction of the mausoleum complex, and the entire project took thirty-nine years to complete^5^,^6^,^7^. In 231 BC, Qin Shi Huang commissioned the building of the city of Liyi (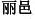) to serve as the base of operations for the construction efforts of the mausoleum^4^,^8^,^9^. Historians estimate it had a population of approximately a hundred thousand individuals, and it was divided into several neighborhoods such as Xi (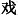) and Jiao (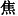)^8^,^9^,^10^. Inscriptions on pottery were found at many sites around the mausoleum, verifying that the inhabitants of Liyi City produced a large quantity of domestic earthenware, brick, tile, and other goods used in both the construction of the tomb and the daily life of the town^11^,^12^. The Chinese historian, Sima Qian (ca. 145-90 BC), wrote that ~700,000 laborers were requisitioned from all over China to toil in the mausoleum over the course of its construction^4^, and that the workers consisted of skilled craftsmen as well as prisoners (captured warriors and/or criminals) and the indebted (people working to pay taxes)^13^,^14^. However, since the Qin Dynasty covered a vast area and included a diverse population of ~20 million people^15^,^16^, the origins of the individuals that comprised this massive labor force are not fully known.

This topic has become an important focus of investigation with archaeologists studying writings on pottery tiles found with the burials of the workers as well as inscriptions on some of the Terracotta soldiers for clues about the people that made them^17^,^18^,^19^. In addition, recent mtDNA evidence suggests the workers had diverse genetic backgrounds from different regions in China^20^. Here we present the first research that isotopically examines the dietary habits of the workers/craftsmen from the public cemetery of Liyi, and the prisoners from the mass grave at Shanren that constructed the famous Qin Shi Huang Mausoleum (see supplementary information for more site details). In addition, these results are compared to other contemporary and earlier sites from across north and south China, and this isotopic information is used in combination with the archaeological evidence and previous mtDNA research^20^ to better understand the diets and possible geographic origins of these individuals.

## Results

The δ^13^C and δ^15^N results of the fauna and humans are presented in Tables S1 and S2 and plotted in Fig. 2. The preservation of bone collagen in human and faunal samples was variable. Of the bones prepared, 161 out of 223 humans and 8 out of 9 faunal samples yielded collagen. The %C, %N and atomic C:N together with collagen yields were used to determine the quality of collagen preservation and assess the effects of diagenesis^21^,^22^. The collagen yields obtained in the present study were low overall, due to the removal of small degraded collagen fragments during the ultrafiltration step^23^, but all samples that produced collagen had C:N values that fell within the range of 2.9–3.6, considered acceptable for stable isotope analysis^24^,^25^.

### Isotopic data for Liyi humans

The Liyi humans (n = 146) show a large variation of isotopic values across the entire population, with the δ^13^C values ranging from −16.3% to −7.0% (mean = −8.7 ± 1.5%) and the δ^15^N values ranging from 6.1% to 12.3% (mean = 10.3 ± 0.7%) (Fig. 2). The majority of individuals have δ^13^C (>−12.0%) and δ^15^N (>9.0%) results indicative of a predominately C_4_ diet that was derived from domestic animal proteins (likely pig, cattle, sheep, dog; Fig. 2). However, several individuals have relatively ^13^C-depleted values, evidence that they consumed a mixed C_3_/C_4_ protein diet. In addition, a single individual (M71) plots well away from the population with a ^13^C-depleted measurement (−16.3%) as well as the most ^15^N-depleted result (6.1%) of all the humans analyzed, suggesting a unique dietary pattern.

### Isotopic data for Shanren humans

The Shanren humans (n = 14) have δ^13^C values that range from −18.2% to −8.5%, with a mean value of −15.4 ± 2.9% and δ^15^N results that range from 7.2% to 10.8%, with a mean value of 8.0 ± 0.6% (Fig. 2). The isotopic results for 12 of the humans (except for M101, M111) cluster in a group (mean values, δ^13^C = −16.4 ± 1.1%; δ^15^N = 8.0 ± 0.6%). The range of δ^13^C results fall within the expected values of a population consuming both C_3_ and C_4_ resources, but are closer to values expected for a purely terrestrial C_3_ protein diet. In contrast, the individuals M101 and M111 have significantly ^13^C-enriched results reflecting that the protein portions of their diets were predominately C_4_ either from plants (M111, low δ^15^N = 7.6%) or from animals that were feeding on C_4_ plants (M101, high δ^15^N = 10.8%).

## Discussion

Substantial research on the agricultural practices of northern China, as well as archaeological evidence from pollen, phytolith and plant flotation studies found that millet agriculture was established in northern China and became the dominant grain for human food or for animal fodder in the late Neolithic^26^,^27^. In this study, the isotopic evidence indicates that millet was the most important crop for the daily diet of the Liyi population during the Qin period. This finding is also supported by previously published isotopic data of contemporary sites from the Guanzhong Plain (an area central to the original Qin state)^4^,^28^ (Table 1). There are no large isotopic differences between these sites (Fig. 3), and nearly all of the north Chinese populations had diets that were predominately based on millets with varying amounts of domestic animal protein consumption (Fig. 3).

The human mean δ^15^N value (10.3 ± 0.7%) indicates a significant consumption of domestic animal protein for the Liyi population as a whole, but individual differences exist (Fig. 2). In comparison with the fauna data, the offsets in the δ^13^C values between the humans and the chicken/crane indicate that these species were not the main sources of protein for the population. In addition, the Liyi population is significantly elevated (one-way ANOVA; p = 0.000) in mean δ^15^N compared to the contemporary period Qin sites of Sunjianantou (δ^15^N = 8.5 ± 1.0%)^29^ and Jianhe (δ^15^N = 8.7 ± 0.5%)^30^, which might indicate that the Liyi people consumed more animal protein in their diet than other Qin sites (Fig. 3). However, due to the small number of faunal samples, we caution against more detailed interpretations.

Historical sources such as *Shijing* (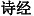)^31^, *Liji* (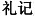)^32^, and *Zhouli* (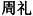)^33^ provide important accounts concerning the diet during the pre-Qin period. Millet, soybean and wheat were discussed as the main crops in the central plains region of China before the Han dynasty. In particular, the *Shuihudi Qin bamboo texts* (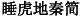), (written during the Qin Dynasty, recording Qin laws and public documents, and excavated from a Qin tomb in Yunmeng County, Hubei Province in 1975)^34^ described that the Qin State stored a large amount of millet in the capital’s granary. In *Shiji* (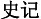), Sima mentioned that the Qin people avoided the consumption of soybean because they viewed it as a crop for the poor and were also uninterested in the planting of wheat^4^. Thus, the isotopic results presented here, that the Qin diet was heavily reliant on millet, are in agreement with the Chinese historical texts. The typical Qin diet was also described as including a number of possible meat sources: cattle, sheep, goat, dog as well as wild boar^35^,^36^,^37^. Sima wrote that there were special shops to sell dried and spiced meats in the city of Xianyang, the capital of the Qin^4^. In addition, *Shiji* (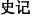) and *Hanshu* (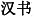) also mentioned that dog meat consumption was prevalent during the Qin and Han Dynasties, since many people made their living by the butchering dogs^4^,^38^.

In contrast to Liyi, the δ^13^C values for the Shanren humans were variable but show a diet reliant on mixed C_3_/C_4_ protein sources, involving the consumption of millet or animals fed millet and possibly rice and/or wheat or wild game over their lifetime (Fig. 2). However, two individuals (M101 and M111) ate predominately C_4_-based foods, and one individual (M113) consumed predominately C_3_-based foods. The δ^15^N values exhibit a narrow range (±1%) for all individuals (except M101), and were similar or only slightly elevated above the local animal species. This possibly suggests these people were consuming less animal protein compared to the population at Liyi which agrees with the fact that these mausoleum workers were of lower status (likely prisoners since buried with iron leg shackles in a mass grave without grave goods; see supplementary section) than the villagers.

Additional evidence to support this possibility comes from historical sources. The *Shuihudi Qin bamboo texts* (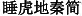) recorded that prisoners were provided food according to their assignments, with workers that built walls or engaged in heavy manual labor receiving ~0.75 kg and ~0.5 kg of rough millet for lunch and dinner, respectively^34^. Since meat and wine were only given to soldiers as a reward, it is unlikely that domestic animal meat was part of a prisoner’s daily diet^34^. However, it is possible that these prisoners could have supplemented their daily diet with wild game. It is interesting to note that only a single individual (M101) was observed to have isotopic values identical to the Liyi population. Thus, it is possible this person could have been a townsperson that was forced to labor in the mausoleum construction as a result of punishment or to pay back a debt. However, if many of the Shanren individuals were not locals and were from other regions of China, then these lower isotopic values would reflect the local environmental food web from where they originated, and this possibility is considered below.

**Figure 1:**
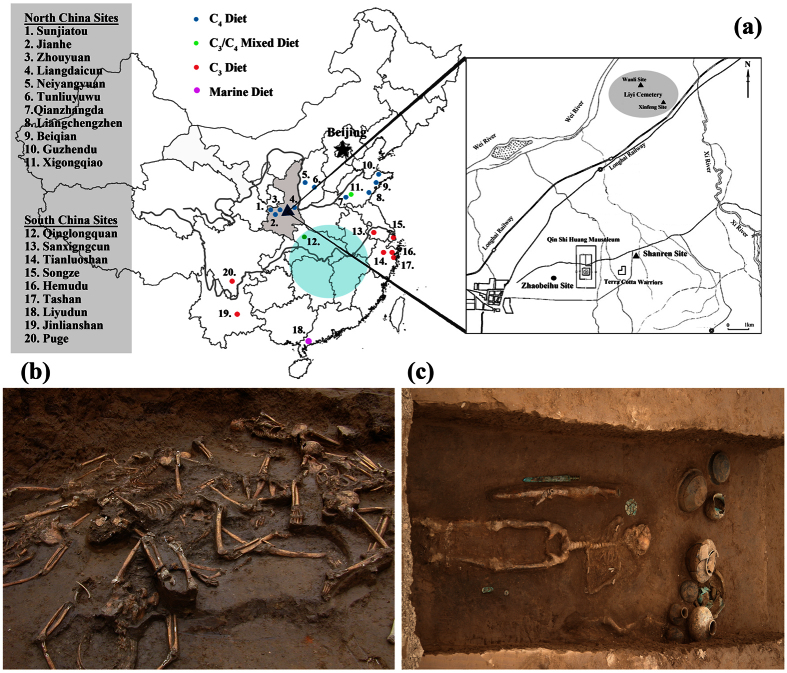
(**a**) Map of mainland China showing the location of the Shanren and Liyi sites in Shaanxi Province (shaded) and the additional archaeological sites mentioned in the text. Shaded circle represents an estimate of the original Chu state from which the mausoleum workers may have originated. (Map was created by the authors, Ying Ma and Weigang Sun, using MATLAB and CorelDRAW) (**b**) Picture showing the excavation of the mausoleum workers from the Shanren site. (**c**) Picture showing the burial of one of the Liyi individuals (Note: the bronze sword and ceramic pottery grave goods). (Pictures b and c were taken by the author, Weigang Sun).

**Figure 2:**
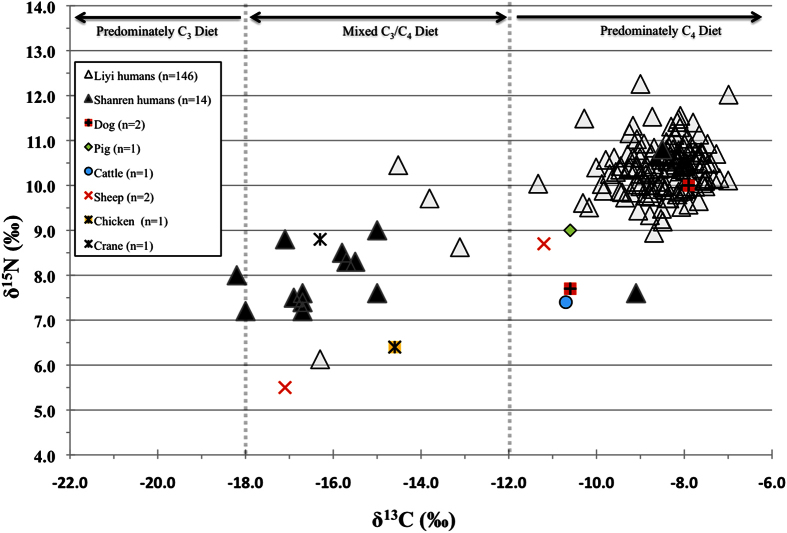
Human and animal δ^13^C and δ^15^N results from the Liyi and Shanren sites.

**Figure 3:**
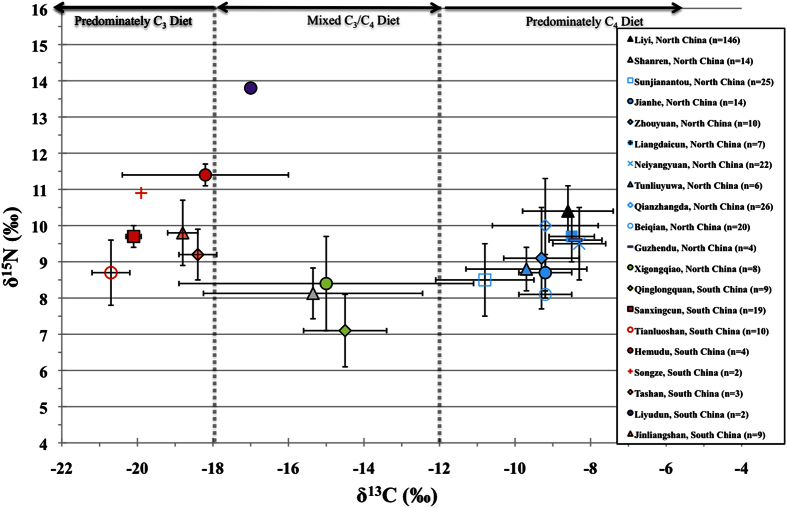
Human δ^13^C and δ^15^N values (mean ± sd) from the Liyi and Shanren sites, and the previous published isotopic results from pre-Qin and Qin populations in north and south China.

While the archaeobotanical remains of rice and wheat have been discovered at many sites in northern China since at least the end of the Neolithic^39^,^40^,^41^,^42^,^43^, archaeological research indicates that distinct dietary differences existed between north (millet) and south (rice) China with the overlapping regions designated as rice-millet blended zones^44^. Current isotopic findings focused on δ^13^C human values support that these general dietary differences existed at the population level between sites in north (C_4_ = millet) and south (C_3_ = rice) China (see Table 1; Figs 1a and 3). These isotopic dietary differences between north and south China can be used as markers to examine the general origins of the Shanren individuals, who died constructing the Mausoleum of Qin Shi Huang. Specifically, evidence from a number of previous isotopic studies in China are used for comparison to the Shanren results (Fig. 3).

All the Western and Eastern Zhou period sites from north China display evidence of significant millet consumption^29^,^30^,^45^,^46^,^47^ (Figs 1a and 3; Table 1). For instance, in Shanxi Province, Pei *et al.*^46^ suggested that the Neiyangyuan people mainly relied on stockbreeding, and the high C_4_ signatures in the δ^13^C values shows that the consumption of millet made a significant contribution to the diet by direct consumption and/or as fodder for their livestock. The isotopic results for people from the eastern Province of Shandong also show a reliance on millet^27^,^48^,^49^,^50^, but the Neolithic Xigongqiao population had a mixed C_3_/C_4_ diet^51^. The hot and humid climate of south China is unfavorable for collagen preservation, and this has resulted in significantly less isotopic research in this region. Since only three relatively contemporary sites from south China exist^52^,^53^,^54^ (Fig. 1a; Table 1), additional south Chinese isotopic results from earlier periods were used^50^,^55^,^56^, and we acknowledge and caution that this time difference between sites is not ideal for direct comparison. Still, by examining the south China δ^13^C results, it can be seen that nearly all populations were consuming C_3_ diets (Figs 1a and 3).

Unfortunately, no grave goods or pottery tiles with identifying information were found in the Shanren mass grave and other research methods and historical sources are needed to determine their origins. When both the carbon and nitrogen results of the Shanren individuals are compared to the pre-Qin site of Qianzhangda in Shandong Province (Table 1; Fig. 3), we see there are no similarities, and that the sites are statistically distinct (one-way ANOVA; p = 0.000 for δ^13^C; p = 0.000 for δ^15^N). However, the Shanren isotopic results are nearly indistinguishable from the much earlier Neolithic site of Xigongqiao (Fig. 3). Given the antiquity of Xigongqiao and that there is only a single pre-Qin site (Qianzhangda) from Shandong, it is difficult determine if the Shanren individuals were originally from the Shandong region, but this is a possibility since archaeobotanical studies have found evidence for populations with rice economies in this region^39^,^41^. Thus, additional isotopic studies focused on Qin period archaeological sites from Shandong are necessary to provide more information.

Compared to the local Liyi population, the Shanren individuals had a mixed diet of millets or animals consuming millets and possibly rice and/or wheat and appear to have consumed less animal products. This decrease in millet consumption appears to correspond with the isotopic results of people from the southern areas China. Given that both the δ^13^C and δ^15^N values for the Shanren humans are similar to the contemporary Qinglongquan site in Hubei Province (Fig. 3) this could suggest that many of the Shanren individuals came from the same general region. However, there are outliers, with the individual M101, having nearly identical isotopic results as the Liyi population, strongly suggesting that he was from the local area. In addition, M111 had a similar δ^13^C value to the Liyi individuals but a significantly lower δ^15^N value. This could suggest two potential possibilities: he was from the Liyi area but consuming millet with little animal protein (a prisoner’s diet) or that he was from another area of northern China that was distinct from the Liyi community.

Excavations at the Zhaobeihu site (another contemporary cemetery consisting of mausoleum prisoners) discovered several fragments of pottery tile with epitaphs that recorded personal information (e.g. names, ranks, birthplaces) about these people and confirm that these prisoners were from distant regions of China^57^,^58^. In particular, these writings show that seventeen individuals were from the eastern region of the Qin State from today’s Shandong Province (n = 10), Jiangsu Province (n = 1), Henan Province (n = 3) and Hebei Province (n = 3)^17^. This suggests that the prisoners were kept as a group and buried together by their rank and general regions of origin (similar language, customs, diet, etc.).

Additional evidence is provided by past ancient DNA analysis. Xu *et al.*^20^ also studied the mtDNA from nineteen workers from the Shanren site, and concluded that many of these people were Hans or minorities from the south of China. Unfortunately, due to different individuals being selected, a direct comparison between the ancient DNA and the isotopic results is only possible for a single individual (M91). M91 had a mixed C_3_/C_4_ δ^13^C signature (−17.1%) that is suggestive of southern China (Fig. 3), and this agrees with the ancient DNA findings that he was genetically related to the southern Han people.^20^ However, since the study of Xu *et al.*^20^ compared the Shanren remains to modern individuals, some skepticism and caution about the results is warranted. There is the possibility that later migrations and admixture between the populations of north and south China could complicate the understanding of the genetics from the archaeological individuals. However, bearing this possibility in mind, our isotopic results suggest that many of the Shanren prisoners had isotopic signatures for mixed C_3_/C_4_ diets found in southern China, and these results are in agreement with the genetic findings of Xu *et al.*^20^.

Finally, according to historical documents that describe the wars and battles of the Qin State^4^,^13^, a southern origin for these Shanren prisoners, forced to construct the Qin Shi Huang Mausoleum, is certainly plausible given the other lines of evidence presented here. Thus, we can further hypothesize that these Shanren individuals were possibly from the rival Chu state, located in modern-day Hubei Province, as well as northern Hunan and southern Anhui Provinces (Fig. 1). In conclusion, while limited and far from perfect, the isotopic results presented here are able to lend some support to the ancient DNA evidence and historical sources, highlighting the benefits of stable isotope studies to document migration in archaeological populations in China.

## Methods

A total of 223 individuals consisting of rib and long-bone fragments were obtained from the Liyi cemetery, from both the “Xinfeng” (n = 166) and “Wanli” sites (n = 57). Fauna samples (pig, dog, cattle, sheep, chicken and crane; n = 9) found in the “Wanli” site during excavation were also collected and analyzed. In addition, human remains (n = 19) consisting of rib and long-bone fragments were sampled from the Shanren site^19^. Additional details about the fauna and humans can be found in Table S1 and S2, respectively.

Collagen samples were prepared following the protocol outlined in Richards and Hedges^59^ modified by using the ultrafiltration method^60^,^61^. Small bone chunks were cleaned by air abrasion and then demineralized at 4 °C in 0.5 M HCl for two weeks. Once demineralized, the samples were rinsed three times with deionized water, and then introduced to a pH = 3 solution and gelatinized at 70 °C for 48 hours. The samples were first filtered with a 5μm EZEE^©^ filter to remove the insoluble residues; then ultrafiltered (Amicon^©^ ultrafilters <30 kDa), and finally the purified collagen was frozen and freeze dried for 2 days. About 0.5 mg of dried collagen was weighed into tin capsules for analysis and each sample was measured in duplicate using a Flash EA 2112 coupled to a Delta XP mass spectrometer (Thermo-Finnigan^®^, Bremen, Germany). Natural abundance of δ^13^C and δ^15^N is expressed as ‘per mil’ (%) with respect to international standards: δ^13^C or δ^15^N = (R_sample_/R_standard_ − 1) * 1000, where R in δ^13^C or δ^15^N is ^13^C/^12^C or ^15^N/^14^N, respectively. Vienna Pee Dee belemnite (VPDB) and atmospheric nitrogen (AIR) were used as the international standards for carbon and nitrogen, respectively. The analytical precision was  ± 0.2% for both δ^13^C and δ^15^N.

## Additional Information

**How to cite this article**: Ma, Y. *et al.* Tracing the locality of prisoners and workers at the Mausoleum of Qin Shi Huang: First Emperor of China (259-210 BC). *Sci. Rep.* **6**, 26731; doi: 10.1038/srep26731 (2016).

## Supplementary Material

Supplementary Information

Supplementary Table S1

Supplementary Table S2

## Figures and Tables

**Table 1:** Summary of isotopic results from the Liyi and Shanren sites as well as from the previous published pre-Qin and Qin populations from north and south China.

Site	#Humans	δ^13^C ± SD (%)	δ^15^N ± SD (%)	Period	Age	Location	Modern Chinese Province	Reference
Sunjianantou	25	−10.8 ± 1.3%	8.5 ± 1.0%	Eastern Zhou	ca. 770−221	North China	Shaanxi	29
Jianhe	14	−9.2 ± 0.7%	8.7 ± 0.5%	Late Eastern Zhou	ca. 476–221	North China	Shaanxi	30
Zhouyuan	10	−9.3 ± 1.0%	9.1 ± 1.4%	Western Zhou	ca. 1046–771	North China	Shaanxi	45
Liangdaicun	7	−8.5 ± 0.6%	9.7 ± 0.7%	Western Zhou	ca. 1046–771	North China	Shaanxi	45
Neiyangyuan	22	−8.3 ± 0.7%	9.5 ± 1.0%	Late Eastern Zhou	ca. 476–221	North China	Shanxi	46
Tunliuyuwu	6	−9.7 ± 1.6%	8.8 ± 0.6%	Late Eastern Zhou	ca. 476–221	North China	Shanxi	47
Qianzhangda	26	−9.2 ± 1.4%	10.0 ± 1.3%	Western Zhou	ca. 1046–771	North China	Shandong	48
Liangchengzhen	15	−9.8 ± 2.0%	–	Neolithic	ca. 2500–2000	North China	Shandong	27
Beiqian	20	−9.2 ± 0.7%	8.1 ± 0.1%	Neolithic	ca. 6100–5500	North China	Shandong	49
Guzhendu	4	−8.4 ± 0.7%	9.6%*	Neolithic	ca. 4300–2500	North China	Shandong	50
Xigongqiao	8	−15.0 ± 3.9%	8.4 ± 1.3%	Neolithic	ca. 5000–4500	North China	Shandong	51
Qinglongquan	9	−14.5 ± 1.1%	7.1 ± 1.0%	Eastern Zhou	ca. 770–221	South China	Hubei	54
Sanxingcun	19	−20.1 ± 0.2%	9.7 ± 0.3%	Early Neolithic	ca. 6500–5500	South China	Jiangsu	55
Tianluoshan	10	−20.7 ± 0.5%	8.7 ± 0.9%	Early Neolithic	ca. 7000–5500	South China	Zhejiang	56
Hemudu	4	−18.2 ± 2.2%	11.4 ± 0.3%	Early Neolithic	ca. 6800–6000	South China	Zhejiang	50
Songze	2	−19.9 ± 0.4%	10.9 ± 1.6%	Early Neolithic	ca. 5800–4900	South China	Shanghai	50
Tashan	3	−18.4 ± 0.5%	9.2 ± 0.7%	Early Neolithic	ca. 5900–5600	South China	Zhejiang	56
Liyudun	2	−17.0 ± 1.3%	13.8 ± 1.4%	Early Neolithic	ca. 7000–6000	South China	Guangdong	62
Jinlianshan	9	−18.8 ± 0.4%	9.8 ± 0.9%	Late Eastern Zhou	ca. 476–221	South China	Yunnan	53
Puge	1	−20.4%	–	Late Eastern Zhou	ca. 476–221	South China	Sichuan	52

^*^Only one sample measured for nitrogen.
