# Supplementary material for: Tracing the locality of prisoners and workers at the Mausoleum of Qin Shi Huang: First Emperor of China (259-210 BC)
Source: Sci Rep. 2016 Jun 2;6:26731. doi: 10.1038/srep26731 (PMC4890548; doi:10.1038/srep26731)
Supplement: Supplementary Information [file srep26731-s1.doc]

**Tracing the locality of prisoners and workers at the Mausoleum of Qin Shi Huang: First Emperor of China (259-210 BC)**

Ying Ma^1^*****, Benjamin T. Fuller^2^, Weigang Sun^3^, Songmei Hu^3^, Liang Chen^4^, Yaowu Hu^2,5^, Michael P. Richards^1,6^

*^1^Department of Human Evolution, Max Planck Institute for Evolutionary Anthropology, 6 Deutcher Platz, D-04103 Leipzig, Germany*

*^2^Department of Archaeology and Anthropology, University of Chinese Academy of Sciences, Beijing 100049, China*

*^3^Shaanxi Provincial Institute of Culture Relics and Archaeology, Xi’an, Shaanxi 710054, P. R. China*

*^4^Institute of Archaeology, Northwest University, Xi’an, Shaanxi 710069, P. R. China*

*^5^Key Laboratory of Vertebrate Evolution and Human Origins of Chinese Academy of Sciences, Institute of Vertebrate Palaeontology and Palaeoanthropology, Chinese Academy of Sciences, Beijing 100044, China*

*^6^Department of Anthropology, University of British Columbia, 6303 NW Marine Drive, Vancouver, BC, V6T 1Z1, Canada*

**KEY WORDS:** Qin Dynasty, Liyi, Shanren, China, Millet, Stable Isotopes

***=Corresponding author**:

Ying Ma

e-mail: [maying_121@126.com](mailto:maying_121@126.com)

**Running Title**: Diet at the Mausoleum of Qin Shi Huang

**Supplementary Information**

**Isotopic analysis and palaeodietary reconstruction**

Carbon (δ^13^C) and nitrogen (δ^15^N) stable isotope ratio analysis is used to investigate a wide range of topics such as subsistence patterns, mobility, health and nutrition, weaning age, and the social stratification of past populations^1-11^. The distinct advantage of this method is that it provides direct evidence about the type of foods that were actually eaten by an individual. Therefore, using stable isotope analysis on archaeological remains, it is possible to examine the dietary contributions of distinctive food groups, and then identify individuals or populations by dietary diversity.

The δ^13^C values can differentiate between C_3_ and C_4_ terrestrial diets in humans and animals. Different photosynthetic pathways used by C_3_ plants (including most vegetables, rice, wheat, and barley), and C_4_ plants (including millet, maize and some tropical grasses), lead to distinct δ^13^C values. For instance, rice has a δ^13^C value averaging about -26‰, whereas the δ^13^C values for millet are about -11.8‰^12^. Experimental evidence from controlled feeding experiments with rodents suggests that isotopes in bone collagen primarily represent a consumer’s protein sources, and that there is ~5‰ δ^13^C shift between diet and consumer bone collagen^13, 14^. The δ^13^C measurement of animal and human remains has been used to successfully reconstruct the spread of C_3_ and C_4_ agriculture and animal husbandry practices from sites across China^12, 15-18^. The δ^15^N values increase by ~3-5‰ per trophic level, principally reflecting the position of an individual in the food chain^2, 19, 20^. Thus, the type and amount of animal protein in the diet can be estimated for individuals when compared to the animals at an archaeological site. An expanded discussion concerning stable isotope ratio analysis is beyond the scope of this work and the following reviews are recommended for greater indepth reading^4, 20, 21, 22^

**Liyi cemetery site**

The Liyi cemetery site is located ~6 km southwest of the Qin Shi Huang Mausoleum and ~40 km from the city of Xi’an, Shaanxi Province, China (Figure 1). From 2007-2010, excavations yielded more than a thousand burials, most of which belonged to the Qin culture and dating from the late phase of the Warring State Period (476-221 BC) through the entire Qin Dynasty (221-207 BC). The majority of graves were aligned W-E, and contained single inhumations in wooden coffins buried with a flexed posture. An examination of the physical anthropology of the skeletons did not reveal evidence of wounds or trauma indicating that the deaths of the individuals were natural (pers. comm., Liang Chen). Most of the burials had associated grave goods: pottery, bronze sacrificial items, bronze swords and iron tools, but some contained high value objects such as jade, silver, and shell. The site was divided into two parts by the construction of a highway, and was excavated by two teams from the Shaanxi Provincial Institute of Culture Relics and Archaeology. The excavators named one side of the site as “Xinfeng” and the other as “Wanli”. More than 20 pottery tiles with the inscription of the Chinese character “Xi” (戏) were unearthed with the burials, supporting that this site was a public cemetery for the people that lived in the Xi district of Liyi.

**Shanren site**

In 2003, a Qin Dynasty kiln (Shanren site) was found just 500 meters away from the Terracotta Army Museum, by a team of archaeologists from the Shaanxi Provincial Institute of Culture Relics and Archaeology (Figure 1a). Unexpectedly, 121 human skeletons, together with a large number of segmental tiles, iron tools and instruments of torture, were unearthed around the kiln site^23^. However, no grave goods or identifying information was found with these individuals. The deceased were buried in a casual manner in a mass grave (some with iron shackles around their legs): stacked layer-upon-layer and thrown into a 10-meter deep pit (Figure 1b). Osteological research on the skeletons revealed that all individuals were adult males between 15-45 years old with the average age at death being 24 years^23^. In addition, evidence of skeletal trauma and arthritis were found on nearly all the remains, indicating that the individuals were engaged in heavy work before death. Thus, the deceased were presumed to be prisoners and/or criminals that worked to build the mausoleum, and likely died suddenly in an accident or were killed, and quickly buried together.

*Faunal isotope data*

The two sheep have very different isotopic values (Figure 2). One individual has a predominately C_4_ diet (δ^13^C = -11.2‰) and the other has a mixed C_3_/C_4_ diet (δ^13^C = -17.1‰). The δ^15^N values are also different between these two sheep (5.5‰ and 8.7‰), and suggest that they were fed different diets or were possibly grazed at different locations near the site. The two birds (chicken, δ^13^C = -14.6‰, δ^15^N = 6.4‰; crane, δ^13^C = -16.3‰, δ^15^N = 8.8‰) have similar δ^13^C results that reflect a mixed C_3_/C_4_ diet, but the nitrogen result of the crane is elevated compared to the chicken (Figure 2). The pig (δ^13^C = -10.6‰), two dogs (δ^13^C = -9.3‰) and cattle (δ^13^C = -10.7‰) all have the most ^13^C-enriched values and were consuming a predominately C_4_ diet of millet (Figure 2). One of the dogs has a similar isotopic value to the Liyi population, and its high δ^15^N value (10.0‰) indicates that it was likely feeding on human refuse^24, 25^. The pig also has an elevated δ^15^N value (9.0‰) that could suggest it was feeding on human waste^26^. The cow has a lower δ^15^N value (7.4‰) that is similar to the other dog (7.7‰), but it is difficult to comment further as we only have a single individual.

**References**

1. van der Merwe, N.J., Vogel, J.C. ^13^C content of human collagen as a measure of prehistoric diet in woodland north America. Nature. 276, 815-816 (1978).

2. Schoeninger, M.J. & DeNiro, M.J. Nitrogen and carbon isotopic composition of bone collagen from marine and terrestrial animals. Geochim. et Cosmochim. Acta. 48, 625-639 (1984).

3. Richards, M.P. et al. Stable isotope analysis reveals variations in human diet at the Poundbury Camp Cemetery Site. J. Archaeol. Sci. 25, 1247-1252 (1998).

4. Katzenberg, M.A. Stable isotope analysis: a tool for studying past diet, demography and life history. (ed. Katzenberg, M.A. & Saunders, S.A.) 305-328 (Biological Anthropology of the Human Skeleton, Wiley-Liss, New York, 2000).

5. Fuller, B.T. et al. Nitrogen balance and δ^15^N: why you’re not what you eat during pregnancy. Rapid Commun. Mass Spectrom. 18, 2889-2896 (2004).

6. Hu, Y., Ambrose, S.H., Wang, C.S. Stable isotopic analysis of human bones from Jiahu site, Henan, China: implications for the transition to agriculture. J. Archaeol. Sci. 33, 1319-1330 (2006).

7. Choy, K. et al. Isotopic evidence of dietary variations and weaning practices in the Gaya Cemetery at Yeanri, Gimhae, South Korea. American J. Physical Anthropol. 142, 74-84 (2010).

8. Bourbou, C. et al. Reconstructing the diets of Greek Byzantine populations (6th–15th centuries AD) using carbon and nitrogen stable isotope ratios. American J. Physical Anthropol. 146, 569-581 (2011).

9. Commendador, A.S. et al. Stable isotope (δ^13^C and δ^15^N) perspective on human diet on Rapa Nui (Easter Island) c.a. 1400-1900 AD. American J. Physical Anthropol. 152, 173-185 (2013).

10. Quintelier, K. et al. Isotopic Examination of Links Between Diet, Social Differentiation, and DISH at the Post-Medieval Carmelite Friary of Aalst, Belgium. American J. Physical Anthropol. 153, 203-213 (2014).

11. Cui, Y. et al. Identification of kinship and occupant status in Mongolian noble

burials of the Yuan Dynasty through a multidisciplinary approach. Phil. Trans. R.

Soc. B. 370, 20130378 (2015).

12. Pechenkina, E.A. et al. Reconstructing northern Chinese Neolithic subsistence practices by isotopic analysis. J. Archaeol. Sci. 32, 1176-1189 (2005).

13. Ambrose, S.H. & Norr, L. Experimental evidence for the relationship of the carbon isotope ratios of whole diet and dietary protein to those of bone collagen and carbonate. (ed. Lambert, J. B. & Grupe, G.) 1-37 (Prehistoric Human Bone: Archaeology at the Molecular Level, Springer Verlag, Berlin, 1993).

14. Tieszen, L.L. & Fagre, T. Effect of diet quality and composition on the isotopic composition of respiratory CO_2_, bone collagen, bioapatite and soft tissue experiments. (ed. Lambert, J.B. & Grupe, G.) 121-155 (Molecular Archaeology of Pre-historic Human Bone, Springer, Berlin, 1993).

15. Hu, Y.W. et al.Stable isotope analysis of humans from Xiaojingshan site: implications for understanding the origin of millet agriculture in China. J. Archaeol. Sci. 35, 2960-2965 (2008).

16. Barton, L. et al. Agricultural origins and the isotopic identity of domestication in northern China. PNAS. 106, 5523-5528 (2009).

17. Atahan, P. et al. Early Neolithic diets at Baijia, Wei River valley, China: Stable carbon and nitrogen isotope analysis of human and faunal remains. J. Archaeol. Sci. 38, 2811-2817 (2011).

18. Chen X. et al. Raising practices of Neolithic livestock evidenced by stable isotope analysis in the Wei River valley, North China. International J. Osteoarchaeol. 10, 1002/oa.2393 (2014).

19. Bocherens, H. & Drucker, D. Trophic level isotopic enrichment of carbon and nitrogen in bone collagen: case studies from recent and ancient terrestrial ecosystems. International J. Osteoarchaeol. 13, 46-53 (2003).

20. Lee-Thorp, J.A. On isotopes and old bones. Archaeometry. 50, 925-950 (2008).

21. Reitsema, L.J. Beyond diet reconstruction: Stable isotope applications to human physiology, health, and nutrition. American J. Human Genetics. 25, 445-456 (2013).

22. Schoeninger, M.J. Stable isotope analyses and the evolution of human diets. Annual Reviews of Anthropology. 43, 413-430 (2014).

23. Shaanxi Provincial Institute of Archaeology & Emperor Qin’s Terracotta Warriors and Horses Museum. Report on Archaeological Researches of the Qin Shihuang Mausoleum Precinct from 2001 to 2003 (Culture Relics Press, Beijing, 2007).

24. Richards, M.P., Fuller, B.T., Molleson, T.I. Stable isotope palaeodietary study of humans and fauna from the multi-period (Iron Age, Viking and Late Medieval) site of Newark Bay, Orkney. J. Archaeol. Sci. 33, 122-131 (2006).

25. Guiry, E.J. Dogs as analogs in stable isotope based human paleodietary recontructoins: A review and considerations for future use. J. Archaeol. Method and Theory. 19, 351-376 (2012).

26. Fuller B.T. et al. Isotopic reconstruction of human diet and animal husbandry practices during the Classical-Hellenistic, Imperial and Byzantine Periods at Sagalassos, Turkey. American J. Physical Anthropol. 149,157-171 (2012).
