# Supplementary material for: Tracing the locality of prisoners and workers at the Mausoleum of Qin Shi Huang: First Emperor of China (259-210 BC)
Source: Sci Rep. 2016 Jun 2;6:26731. doi: 10.1038/srep26731 (PMC4890548; doi:10.1038/srep26731)
Supplement: Supplementary Table S1 [file srep26731-s2.doc]

**Tracing the locality of prisoners and workers at the Mausoleum of Qin Shi Huang: First Emperor of China (259-210 BC)**

Ying Ma^1^*****, Benjamin T. Fuller^2^, Weigang Sun^3^, Songmei Hu^3^, Liang Chen^4^, Yaowu Hu^2,5^, Michael P. Richards^1,6^

*^1^Department of Human Evolution, Max Planck Institute for Evolutionary Anthropology, 6 Deutcher Platz, D-04103 Leipzig, Germany*

*^2^Department of Archaeology and Anthropology, University of Chinese Academy of Sciences, Beijing 100049, China*

*^3^Shaanxi Provincial Institute of Culture Relics and Archaeology, Xi’an, Shaanxi 710054, P. R. China*

*^4^Institute of Archaeology, Northwest University, Xi’an, Shaanxi 710069, P. R. China*

*^5^Key Laboratory of Vertebrate Evolution and Human Origins of Chinese Academy of Sciences, Institute of Vertebrate Palaeontology and Palaeoanthropology, Chinese Academy of Sciences, Beijing 100044, China*

*^6^Department of Anthropology, University of British Columbia, 6303 NW Marine Drive, Vancouver, BC, V6T 1Z1, Canada*

**KEY WORDS:** Qin Dynasty, Liyi, Shanren, China, Millet, Stable Isotopes

***=Corresponding author**:

Ying Ma

e-mail: [maying_121@126.com](mailto:maying_121@126.com)

**Running Title**: Diet at the Mausoleum of Qin Shi Huang

**Supplementary Table S1**

Table S1. Isotopic results and sample information for all fauna from the Liyi cemetery (Wanli), Shaanxi Province, China.

| **Site** | **ID #** | **Taxon** | **Element** | **%Yield** | **δ^13^C (‰)** | **δ^15^N (‰)** | **%C** | **%N** | **C:N** |
| --- | --- | --- | --- | --- | --- | --- | --- | --- | --- |
| Liyi (Wanli) | M122 | Pig | Scapula | 0.3 | -10.6 | 9.0 | 38.9 | 13.8 | 3.3 |
| Liyi (Wanli) | M24 | Dog | Humerus | 3.4 | -10.6 | 7.7 | 45.1 | 16.4 | 3.2 |
| Liyi (Wanli) | M111 | Dog | Cubitus | 1.1 | -7.9 | 10.0 | 44.3 | 15.9 | 3.3 |
| Liyi (Wanli) | M122 | Sheep | Vertebra | 0.4 | -11.2 | 8.7 | 45.1 | 15.8 | 3.3 |
| Liyi (Wanli) | M37 | Sheep | Scapula | 4.8 | -17.1 | 5.5 | 47.1 | 17 | 3.2 |
| Liyi (Wanli) | M122 | Chicken | Tibia | 1.0 | -14.6 | 6.4 | 44.8 | 15.8 | 3.3 |
| Liyi (Wanli) | M122 | Crane | Tibia | 3.1 | -16.3 | 8.8 | 45.5 | 16.4 | 3.2 |
| Liyi (Wanli) | M111 | Cattle | Tibia | 2.9 | -10.7 | 7.4 | 42.5 | 15.3 | 3.2 |
| **The sample below failed to produced collagen** | | | | | | | | | |
| Liyi (Wanli) | M85 | Horse | Phalanx | -- | -- | -- | -- | -- | -- |
