# Supplementary material for: Tracing the locality of prisoners and workers at the Mausoleum of Qin Shi Huang: First Emperor of China (259-210 BC)
Source: Sci Rep. 2016 Jun 2;6:26731. doi: 10.1038/srep26731 (PMC4890548; doi:10.1038/srep26731)
Supplement: Supplementary Table S2 [file srep26731-s3.doc]

**Tracing the locality of prisoners and workers at the Mausoleum of Qin Shi Huang: First Emperor of China (259-210 BC)**

Ying Ma^1^*****, Benjamin T. Fuller^2^, Weigang Sun^3^, Songmei Hu^3^, Liang Chen^4^, Yaowu Hu^2,5^, Michael P. Richards^1,6^

*^1^Department of Human Evolution, Max Planck Institute for Evolutionary Anthropology, 6 Deutcher Platz, D-04103 Leipzig, Germany*

*^2^Department of Archaeology and Anthropology, University of Chinese Academy of Sciences, Beijing 100049, China*

*^3^Shaanxi Provincial Institute of Culture Relics and Archaeology, Xi’an, Shaanxi 710054, P. R. China*

*^4^Institute of Archaeology, Northwest University, Xi’an, Shaanxi 710069, P. R. China*

*^5^Key Laboratory of Vertebrate Evolution and Human Origins of Chinese Academy of Sciences, Institute of Vertebrate Palaeontology and Palaeoanthropology, Chinese Academy of Sciences, Beijing 100044, China*

*^6^Department of Anthropology, University of British Columbia, 6303 NW Marine Drive, Vancouver, BC, V6T 1Z1, Canada*

**KEY WORDS:** Qin Dynasty, Liyi, Shanren, China, Millet, Stable Isotopes

***=Corresponding author**:

Ying Ma

e-mail: [maying_121@126.com](mailto:maying_121@126.com)

**Running Title**: Diet at the Mausoleum of Qin Shi Huang

**Supplementary Table S2**

Table S2. Isotopic results and sample information for all humans from the Shanren and Liyi (Xinfeng & Wanli) sites, Shaanxi Province, China.

| **Site** | **ID #** | **Period** | **Element** | **Sex** | **Age** | **%Yield** | **δ^13^C (‰)** | **δ^15^N (‰)** | **%C** | **%N** | **C:N** | **Pathology** | **Grave Goods** |
| --- | --- | --- | --- | --- | --- | --- | --- | --- | --- | --- | --- | --- | --- |
| Shanren | M91 | Qin  Ca. 221-207 BC | Tibia | Male | 18-22 | 1.0 | -17.1 | 8.8 | 43.9 | 15.5 | 3.3 | Periodontitis, Cubitus fracture | None |
| Shanren | M92 | Qin  Ca. 221-207 BC | Humerus | Male | 25 | 0.4 | -16.9 | 7.5 | 21.5 | 7.5 | 3.4 | Myositis ossificans,  Dental caries | None |
| Shanren | M93 | Qin  Ca. 221-207 BC | Humerus | Male | 18-20 | 1.0 | -16.7 | 7.4 | 43.2 | 15.4 | 3.3 | Periodontitis | None |
| Shanren | M96 | Qin  Ca. 221-207 BC | Tibia | Male | 17-18 | 0.4 | -15.7 | 8.3 | 40.8 | 14.2 | 3.3 | Periodontitis | None |
| Shanren | M99 | Qin  Ca. 221-207 BC | Femur | Male | 25 | 1.2 | -16.7 | 7.6 | 44.4 | 15.9 | 3.3 | Periodontitis  Impacted tooth | None |
| Shanren | M112 | Qin  Ca. 221-207 BC | Humerus | Male | 40 | 0.7 | -16.7 | 7.2 | 27.4 | 9.7 | 3.3 | Periodontitis  Periapical abscess | None |
| Shanren | M101 | Qin  Ca. 221-207 BC | Tibia | Male | 20-25 | 0.8 | -8.5 | 10.8 | 42.7 | 14.8 | 3.4 | Periodontitis | None |
| Shanren | M102 | Qin  Ca. 221-207 BC | Rib | Male | 17-19 | 0.4 | -18.0 | 7.2 | 30.6 | 10.6 | 3.4 | Unknown | None |
| Shanren | M103 | Qin  Ca. 221-207 BC | Tibia | Male | Adult | 0.2 | -15.8 | 8.5 | 17.8 | 6.4 | 3.3 | Unknown | None |
| Shanren | M107 | Qin  Ca. 221-207 BC | Tibia | Male | 16-18 | 0.3 | -15.0 | 7.6 | 37.9 | 13 | 3.4 | Impacted tooth | None |
| Shanren | M108 | Qin  Ca. 221-207 BC | Rib | Male | 18-22 | 0.3 | -15.5 | 8.3 | 20.7 | 7.7 | 3.1 | Periodontitis | None |
| Shanren | M109 | Qin  Ca. 221-207 BC | Femur | Male | 25 | 1.3 | -15.0 | 9.0 | 28.8 | 10.7 | 3.1 | Periodontitis  Impacted tooth | None |
| Shanren | M111 | Qin  Ca. 221-207 BC | Femur | Male | 20-22 | 1.6 | -9.1 | 7.6 | 36.7 | 13.5 | 3.2 | Periodontitis | None |
| Shanren | M113 | Qin  Ca. 221-207 BC | Tibia | Male | 18-20 | 0.4 | -18.2 | 8.0 | 30.8 | 10.9 | 3.3 | Unknown | None |
| Liyi (Xinfeng) | M66 | Qin  Ca. 221-207 BC | Tibia | Male | 20-25 | 0.2 | -8.1 | 10.4 | 14.7 | 5.3 | 3.2 | Unknown | 4 common pottery,  1 bronze belt hook |
| Liyi (Xinfeng) | M71 | Qin  Ca. 221-207 BC | Tibia | Male | 40-44 | 0.6 | -16.3 | 6.1 | 38.1 | 13.9 | 3.2 | Unknown | 7 common pottery |
| Liyi (Xinfeng) | M72 | Qin  Ca. 221-207 BC | Femur | Female | 45-50 | 0.7 | -8.0 | 10.4 | 41.5 | 15.1 | 3.2 | Unknown | 2 common pottery,  2 bronze vessels |
| Liyi (Xinfeng) | M73 | Qin  Ca. 221-207 BC | Tibia | Male | 30 | 0.7 | -10.3 | 11.5 | 33.8 | 11.7 | 3.4 | Unknown | 2 bronze vessels  2 jade objects |
| Liyi (Xinfeng) | M82 | Qin  Ca. 221-207 BC | Tibia | Female | 24-26 | 0.2 | -9.5 | 9.9 | 31.3 | 11.2 | 3.3 | Unknown | 8 common pottery,  8 bronze vessels,  1 bone object |
| Liyi (Xinfeng) | M94 | Qin  Ca. 221-207 BC | Tibia | Female | 17-19 | 0.2 | -9.0 | 12.3 | 13.1 | 4.7 | 3.2 | Unknown | 7 common pottery |
| Liyi (Xinfeng) | M117 | Qin  Ca. 221-207 BC | Tibia | Female | >60 | 0.6 | -8.0 | 10.5 | 30.7 | 11.3 | 3.2 | Unknown | 9 common pottery |
| Liyi (Xinfeng) | M131 | Qin  Ca. 221-207 BC | Tibia | Male | Adult | 1.0 | -8.3 | 10.7 | 30.0 | 10.9 | 3.2 | Unknown | None |
| Liyi (Xinfeng) | M136 | Qin  Ca. 221-207 BC | Tibia | Female | >60 | 1.2 | -7.7 | 11.1 | 37.7 | 13.5 | 3.2 | Unknown | 3 common pottery |
| Liyi (Xinfeng) | M140 | Qin  Ca. 221-207 BC | Humerus | Female | 17-19 | 0.5 | -7.0 | 10.1 | 30.8 | 11.2 | 3.2 | Unknown | 9 common pottery,  1 iron object |
| Liyi (Xinfeng) | M143 | Qin  Ca. 221-207 BC | Femur | Female | 31-34 | 1.8 | -8.7 | 9.9 | 28.8 | 10.2 | 3.3 | Unknown | None |
| Liyi (Xinfeng) | M145 | Qin  Ca. 221-207 BC | Humerus | Female | 40 | 8.1 | -7.9 | 10.5 | 33.4 | 12.2 | 3.2 | Unknown | 7 common pottery,  1 bronze vessel |
| Liyi (Xinfeng) | M147 | Qin  Ca. 221-207 BC | Tibia | Female | >60 | 2.1 | -8.1 | 9.5 | 24.0 | 8.6 | 3.2 | Unknown | 8 common pottery,  3 bronze vessels |
| Liyi (Xinfeng) | M154 | Qin  Ca. 221-207 BC | Tibia | Female | Adult | 10.3 | -9.8 | 10.6 | 36.6 | 13.4 | 3.2 | Unknown | 5 common pottery,  2 bronze vessels |
| Liyi (Xinfeng) | M157 | Qin  Ca. 221-207 BC | Femur | Female | 50 | 4.9 | -8.5 | 10.1 | 40.5 | 14.8 | 3.2 | Unknown | 5 common pottery |
| Liyi (Xinfeng) | M161 | Qin  Ca. 221-207 BC | Tibia | Female | 51-60 | 3.1 | -10.0 | 10.4 | 31.4 | 11.2 | 3.3 | Unknown | 7 common pottery |
| Liyi (Xinfeng) | M164 | Qin  Ca. 221-207 BC | Femur | Male | 51-60 | 6.6 | -8.8 | 9.8 | 21.4 | 7.7 | 3.2 | Unknown | 2 bronze vessels |
| Liyi (Xinfeng) | M165 | Qin  Ca. 221-207 BC | Tibia | Female | 25-30 | 3.4 | -7.7 | 9.7 | 37.8 | 13.5 | 3.3 | Unknown | None |
| Liyi (Xinfeng) | M177 | Qin  Ca. 221-207 BC | Femur | Male | 25-30 | 0.6 | -8.7 | 11.5 | 41.2 | 14.6 | 3.3 | Unknown | 11 common pottery,  2 bronze vessels,  1 iron object,  1 bone object,  1 stone object,  1 jade object |
| Liyi (Xinfeng) | M182 | Qin  Ca. 221-207 BC | Tibia | ? | ? | 8.9 | -9.0 | 10.0 | 35.3 | 12.8 | 3.2 | Unknown | None |
| Liyi (Xinfeng) | M205 | Qin  Ca. 221-207 BC | Femur | Female | 30 | 5.5 | -14.5 | 10.5 | 40.4 | 14.7 | 3.2 | Unknown | 2 bronze vessels,  4 jade objects, |
| Liyi (Xinfeng) | M213 | Qin  Ca. 221-207 BC | Tibia | Male | 45-50 | 2.8 | -8.4 | 10.7 | 40.5 | 14.6 | 3.2 | Unknown | 7 common pottery,  1 bronze vessel |
| Liyi (Xinfeng) | M221 | Qin  Ca. 221-207 BC | Tibia | Male | Adult | 1.4 | -8.3 | 11.0 | 24.8 | 9 | 3.2 | Unknown | 7 bronze vessels,  2 silver loops |
| Liyi (Xinfeng) | M228 | Qin  Ca. 221-207 BC | Femur | Male | 31-34 | 1.9 | -9.5 | 10.4 | 41.3 | 14.7 | 3.3 | Unknown | 1 bronze vessel |
| Liyi (Xinfeng) | M231 | Qin  Ca. 221-207 BC | Tibia | ? | ? | 0.3 | -7.6 | 10.7 | 25.9 | 9.5 | 3.2 | Unknown | Unknown |
| Liyi (Xinfeng) | M244 | Qin  Ca. 221-207 BC | Tibia | Female | 20-23 | 0.4 | -8.2 | 9.8 | 43.0 | 15.5 | 3.2 | Unknown | 7 common pottery,  2 bronze vessels,  1 iron object,  1 bone object |
| Liyi (Xinfeng) | M271 | Qin  Ca. 221-207 BC | Tibia | Male | 60 | 0.3 | -7.5 | 10.0 | 24.5 | 8.9 | 3.2 | Unknown | 3 common pottery |
| Liyi (Xinfeng) | M274 | Qin  Ca. 221-207 BC | Tibia | Male | Adult | 0.2 | -7.5 | 10.1 | 24.2 | 8.8 | 3.2 | Unknown | 1 common pottery,  2 bronze vessels |
| Liyi (Xinfeng) | M280 | Qin  Ca. 221-207 BC | Tibia | Male | Adult | 0.3 | -8.0 | 10.8 | 39.0 | 13.8 | 3.3 | Unknown | 2 common pottery,  1 bronze vessel,  1 iron object |
| Liyi (Xinfeng) | M281 | Qin  Ca. 221-207 BC | Femur | Female | 35 | 0.2 | -8.5 | 10.6 | 33.7 | 12.1 | 3.3 | Unknown | 1 bronze vessel |
| Liyi (Xinfeng) | M283 | Qin  Ca. 221-207 BC | Tibia | Female | Adult | 0.4 | -8.8 | 10.0 | 21.6 | 7.7 | 3.3 | Unknown | 1 bronze vessel |
| Liyi (Xinfeng) | M284 | Qin  Ca. 221-207 BC | Humerus | Male | 35-39 | 0.3 | -7.8 | 10.0 | 26.7 | 9.6 | 3.2 | Unknown | 1 bronze vessel |
| Liyi (Xinfeng) | M288 | Qin  Ca. 221-207 BC | Tibia | Male | >60 | 0.7 | -8.5 | 10.7 | 23.9 | 8.5 | 3.3 | Unknown | 1 common pottery |
| Liyi (Xinfeng) | M290 | Qin  Ca. 221-207 BC | Humerus | Male | >60 | 0.0 | -8.1 | 11.5 | 42.1 | 14.6 | 3.4 | Unknown | None |
| Liyi (Xinfeng) | M292 | Qin  Ca. 221-207 BC | Femur | Male | Adult | 0.5 | -7.9 | 10.3 | 34.8 | 12.4 | 3.3 | Unknown | None |
| Liyi (Xinfeng) | M291 | Qin  Ca. 221-207 BC | Tibia | ? | ? | 0.6 | -9.4 | 9.7 | 10.3 | 3.6 | 3.4 | Unknown | Unknown |
| Liyi (Xinfeng) | M293 | Qin  Ca. 221-207 BC | Tibia | Male | 25 | 0.4 | -8.0 | 10.3 | 32.6 | 11.6 | 3.3 | Unknown | None |
| Liyi (Xinfeng) | M302 | Qin  Ca. 221-207 BC | Femur | Female | 40 | 0.3 | -9.1 | 10.0 | 18.0 | 6.2 | 3.4 | Unknown | None |
| Liyi (Xinfeng) | M305 | Qin  Ca. 221-207 BC | Tibia | Male | >60 | 0.3 | -7.8 | 10.6 | 37.9 | 13.6 | 3.3 | Unknown | None |
| Liyi (Xinfeng) | M309 | Qin  Ca. 221-207 BC | Rib | Male | >60 | 1.1 | -8.0 | 10.4 | 41.4 | 15 | 3.2 | Unknown | 1 bronze vessel |
| Liyi (Xinfeng) | M314 | Qin  Ca. 221-207 BC | Tibia | Female | 25-30 | 0.6 | -9.3 | 11.2 | 35.2 | 12.6 | 3.3 | Unknown | 2 common pottery,  1 bronze vessel,  1 silver object |
| Liyi (Xinfeng) | M325 | Qin  Ca. 221-207 BC | Humerus | Male | 30 | 0.3 | -9.9 | 10.0 | 24.5 | 8.6 | 3.3 | Unknown | 4 common pottery |
| Liyi (Xinfeng) | M326 | Qin  Ca. 221-207 BC | Tibia | Male | 50 | 0.1 | -9.7 | 10.3 | 25.1 | 8.5 | 3.5 | Unknown | 6 common pottery |
| Liyi (Xinfeng) | M331 | Qin  Ca. 221-207 BC | Tibia | Male | 45-50 | 0.8 | -8.1 | 10.3 | 28.5 | 10.3 | 3.2 | Unknown | 1 bronze vessel |
| Liyi (Xinfeng) | M334 | Qin  Ca. 221-207 BC | Femur | Female | 20 | 0.2 | -8.9 | 10.4 | 37.3 | 12.9 | 3.4 | Unknown | 1 bronze vessel |
| Liyi (Xinfeng) | M335 | Qin  Ca. 221-207 BC | Femur | Female | 50 | 0.2 | -9.3 | 10.4 | 19.5 | 6.7 | 3.4 | Unknown | 1 bronze vessel |
| Liyi (Xinfeng) | M337 | Qin  Ca. 221-207 BC | Tibia | ? | ? | 0.1 | -9.4 | 9.9 | 31.2 | 10.7 | 3.4 | Unknown | None |
| Liyi (Xinfeng) | M338 | Qin  Ca. 221-207 BC | Tibia | Male | 50 | 1.5 | -8.8 | 10.3 | 43.2 | 15.5 | 3.3 | Unknown | None |
| Liyi (Xinfeng) | M339 | Qin  Ca. 221-207 BC | Tibia | Male | 25-30 | 0.4 | -8.6 | 10.0 | 24.0 | 8.4 | 3.3 | Unknown | None |
| Liyi (Xinfeng) | M341 | Qin  Ca. 221-207 BC | Tibia | Male | 20-25 | 0.6 | -8.4 | 10.0 | 24.5 | 8.7 | 3.3 | Unknown | None |
| Liyi (Xinfeng) | M342 | Qin  Ca. 221-207 BC | Tibia | ? | ? | 0.2 | -8.5 | 9.5 | 18.2 | 6.3 | 3.4 | Unknown | Unknown |
| Liyi (Xinfeng) | M344 | Qin  Ca. 221-207 BC | Femur | Female | 40 | 0.2 | -10.3 | 9.6 | 25.8 | 8.3 | 3.7 | Unknown | 1 bronze vessel |
| Liyi (Xinfeng) | M346 | Qin  Ca. 221-207 BC | Femur | Male | 50 | 1.1 | -10.2 | 9.5 | 31.3 | 11.3 | 3.2 | Unknown | 1 bronze vessel |
| Liyi (Xinfeng) | M349 | Qin  Ca. 221-207 BC | Tibia | Male | 50-55 | 0.3 | -7.6 | 10.5 | 37.8 | 13.1 | 3.4 | Unknown | 1 bronze vessel |
| Liyi (Xinfeng) | M355 | Qin  Ca. 221-207 BC | Tibia | Female | 18-19 | 0.1 | -9.6 | 10.6 | 39.6 | 13.6 | 3.4 | Unknown | 1 iron object |
| Liyi (Xinfeng) | M444 | Qin  Ca. 221-207 BC | Tibia | Female | 45-50 | 0.2 | -8.0 | 9.9 | 25.2 | 8.9 | 3.3 | Unknown | None |
| Liyi (Xinfeng) | M445 | Qin  Ca. 221-207 BC | Tibia | Male | 45 | 1.1 | -8.1 | 10.8 | 32.5 | 11.8 | 3.2 | Unknown | None |
| Liyi (Xinfeng) | M453 | Qin  Ca. 221-207 BC | Humerus | Male | 45-50 | 0.2 | -8.1 | 10.6 | 34.7 | 12.4 | 3.3 | Unknown | 1 iron object |
| Liyi (Xinfeng) | M454 | Qin  Ca. 221-207 BC | Femur | Female | 45-50 | 0.7 | -8.0 | 10.9 | 37.6 | 13.7 | 3.2 | Unknown | 3 common pottery,  1 bronze vessel |
| Liyi (Xinfeng) | M455 | Qin  Ca. 221-207 BC | Tibia | ? | ? | 0.7 | -8.3 | 11.3 | 37.7 | 13.9 | 3.2 | Unknown | None |
| Liyi (Xinfeng) | M458 | Qin  Ca. 221-207 BC | Humerus | ? | ? | 0.3 | -7.5 | 10.5 | 30.7 | 11.1 | 3.2 | Unknown | 1 iron object |
| Liyi (Xinfeng) | M459 | Qin  Ca. 221-207 BC | Tibia | ? | ? | 0.8 | -9.6 | 10.3 | 31.0 | 11.2 | 3.2 | Unknown | 1 bronze vessel |
| Liyi (Xinfeng) | M465 | Qin  Ca. 221-207 BC | Humerus | ? | ? | 0.3 | -9.1 | 9.4 | 34.3 | 12.7 | 3.1 | Unknown | None |
| Liyi (Xinfeng) | M469 | Qin  Ca. 221-207 BC | Femur | Male | Adult | 0.5 | -9.1 | 10.1 | 30.2 | 11.1 | 3.2 | Unknown | 1 bronze vessel |
| Liyi (Xinfeng) | M477 | Qin  Ca. 221-207 BC | Tibia | ? | ? | 0.9 | -7.8 | 11.4 | 31.4 | 10.7 | 3.2 | Unknown | None |
| Liyi (Xinfeng) | M480 | Qin  Ca. 221-207 BC | Femur | Male | Adult | 1.3 | -7.4 | 10.5 | 37.4 | 13.8 | 3.2 | Unknown | None |
| Liyi (Xinfeng) | M484 | Qin  Ca. 221-207 BC | Tibia | Male | 45-50 | 0.7 | -7.2 | 10.2 | 41.6 | 15.4 | 3.2 | Unknown | None |
| Liyi (Xinfeng) | M486 | Qin  Ca. 221-207 BC | Rib | Male | 40-44 | 0.6 | -7.6 | 10.5 | 31.2 | 11.3 | 3.2 | Unknown | None |
| Liyi (Xinfeng) | M489 | Qin  Ca. 221-207 BC | Tibia | ? | ? | 0.4 | -9.2 | 10.4 | 35.8 | 12.9 | 3.2 | Unknown | None |
| Liyi (Xinfeng) | M491 | Qin  Ca. 221-207 BC | Humerus | ? | ? | 0.5 | -7.6 | 10.4 | 34.9 | 12.8 | 3.2 | Unknown | None |
| Liyi (Xinfeng) | M518 | Qin  Ca. 221-207 BC | Femur | Female | >60 | 0.4 | -8.5 | 10.7 | 40.2 | 14.7 | 3.2 | Unknown | 1 bronze vessel |
| Liyi (Xinfeng) | M519 | Qin  Ca. 221-207 BC | Rib | Female | 45-50 | 0.2 | -9.2 | 11.3 | 37.3 | 13.4 | 3.3 | Unknown | 1 bronze vessel |
| Liyi (Xinfeng) | M532 | Qin  Ca. 221-207 BC | Femur | Female | >60 | 0.5 | -8.5 | 10.0 | 35.2 | 12.8 | 3.2 | Unknown | 1 common pottery |
| Liyi (Xinfeng) | M540 | Qin  Ca. 221-207 BC | Humerus | Female | 40 | 0.9 | -8.9 | 10.9 | 32.8 | 11.9 | 3.2 | Unknown | 1 bronze vessel |
| Liyi (Xinfeng) | M542 | Qin  Ca. 221-207 BC | Tibia | Female | Adult | 0.2 | -8.0 | 10.0 | 33.6 | 11.9 | 3.3 | Unknown | None |
| Liyi (Xinfeng) | M554 | Qin  Ca. 221-207 BC | Tibia | Female | 50 | 0.8 | -8.4 | 10.5 | 37.3 | 13.3 | 3.3 | Unknown | None |
| Liyi (Xinfeng) | M561 | Qin  Ca. 221-207 BC | Femur | Female | >60 | 1.0 | -7.3 | 10.7 | 32.7 | 11.9 | 3.2 | Unknown | None |
| Liyi (Xinfeng) | M564 | Qin  Ca. 221-207 BC | Tibia | Female | Adult | 0.6 | -9.0 | 10.6 | 36.6 | 12.8 | 3.3 | Unknown | 1 iron object |
| Liyi (Xinfeng) | M566 | Qin  Ca. 221-207 BC | Humerus | Female | >60 | 1.7 | -8.4 | 10.8 | 38.5 | 13.9 | 3.2 | Unknown | 3 common pottery |
| Liyi (Xinfeng) | M583 | Qin  Ca. 221-207 BC | Rib | Female | Adult | 0.2 | -8.3 | 9.7 | 23.5 | 8.5 | 3.2 | Unknown | 1 bronze vessel |
| Liyi (Xinfeng) | M572 | Qin  Ca. 221-207 BC | Tibia | ? | ? | 1.4 | -8.7 | 10.8 | 40.5 | 14.7 | 3.2 | Unknown | 1 common pottery |
| Liyi (Xinfeng) | M575 | Qin  Ca. 221-207 BC | Femur | Male | 25-30 | 0.6 | -9.5 | 10.4 | 38.0 | 13.6 | 3.3 | Unknown | 2 common pottery |
| Liyi (Xinfeng) | M586 | Qin  Ca. 221-207 BC | Femur | Female | Adult | 0.5 | -7.7 | 10.7 | 39.1 | 13.9 | 3.3 | Unknown | 4 common pottery |
| Liyi (Xinfeng) | M588 | Qin  Ca. 221-207 BC | Tibia | Male | 40-45 | 0.4 | -8.6 | 10.6 | 39.9 | 14.2 | 3.3 | Unknown | 1 iron object |
| Liyi (Xinfeng) | M590 | Qin  Ca. 221-207 BC | Femur | Male | >60 | 0.6 | -9.2 | 9.8 | 36.2 | 13.1 | 3.2 | Unknown | 1 bronze vessel |
| Liyi (Xinfeng) | M591 | Qin  Ca. 221-207 BC | Tibia | ? | ? | 0.3 | -8.0 | 9.9 | 24.9 | 8.9 | 3.3 | Unknown | None |
| Liyi (Xinfeng) | M605 | Qin  Ca. 221-207 BC | Tibia | ? | ? | 0.8 | -8.2 | 10.5 | 27.9 | 10.1 | 3.2 | Unknown | None |
| Liyi (Xinfeng) | M641 | Qin  Ca. 221-207 BC | Femur | ? | ? | 0.4 | -8.4 | 10.7 | 36.2 | 12.9 | 3.3 | Unknown | None |
| Liyi (Xinfeng) | M678 | Qin  Ca. 221-207 BC | Tibia | ? | ? | 1.2 | -8.1 | 11.6 | 40.7 | 14.7 | 3.2 | Unknown | 14 common pottery,  4 bronze vessels |
| Liyi (Xinfeng) | M701 | Qin  Ca. 221-207 BC | Tibia | ? | ? | 0.7 | -9.3 | 10.7 | 39.0 | 14.0 | 3.3 | Unknown | None |
| Liyi (Xinfeng) | M704 | Qin  Ca. 221-207 BC | Femur | ? | ? | 1.4 | -8.2 | 11.1 | 38.3 | 13.7 | 3.3 | Unknown | 6 common pottery |
| Liyi (Xinfeng) | M709 | Qin  Ca. 221-207 BC | Humerus | ? | ? | 0.8 | -8.2 | 11.4 | 38.4 | 13.4 | 3.3 | Unknown | 6 common pottery |
| Liyi (Xinfeng) | M714 | Qin  Ca. 221-207 BC | Tibia | ? | ? | 0.0 | -9.1 | 10.4 | 37.0 | 12.9 | 3.4 | Unknown | None |
| Liyi (Xinfeng) | M715 | Qin  Ca. 221-207 BC | Femur | ? | ? | 0.2 | -9.1 | 11.0 | 35.7 | 12.8 | 3.3 | Unknown | None |
| Liyi (Xinfeng) | M718 | Qin  Ca. 221-207 BC | Rib | ? | ? | 0.8 | -8.6 | 10.7 | 28.1 | 10.0 | 3.3 | Unknown | 3 common pottery |
| Liyi (Wanli) | M3 | Qin  Ca. 221-207 BC | Humerus | ? | ? | 0.9 | -7.9 | 10.7 | 42.4 | 15.0 | 3.3 | Unknown | 1 bronze belt hook  7 common pottery |
| Liyi (Wanli) | M4 | Qin  Ca. 221-207 BC | Tibia | Male | 22-24 | 0.5 | -8.6 | 10.3 | 42.1 | 15.4 | 3.2 | Unknown | 1 bronze mirror  1 common pottery |
| Liyi (Wanli) | M5 | Qin  Ca. 221-207 BC | Tibia | Female | 40-44 | 0.3 | -8.1 | 11.3 | 41.2 | 14.3 | 3.4 | Unknown | 1 jade quadrate stamp |
| Liyi (Wanli) | M6 | Qin  Ca. 221-207 BC | Tibia | Female | 30-35 | 0.2 | -8.5 | 9.2 | 26.2 | 9.4 | 3.3 | Unknown | None |
| Liyi (Wanli) | M7 | Qin  Ca. 221-207 BC | Humerus | Female | 30-35 | 0.2 | -8.1 | 10.4 | 27.3 | 9.6 | 3.3 | Unknown | 1 bronze loop  1 iron belt hook  1 common pottery |
| Liyi (Wanli) | M9 | Qin  Ca. 221-207 BC | Tibia | Male | 30-35 | 0.6 | -8.4 | 9.7 | 40.7 | 14.9 | 3.2 | Unknown | None |
| Liyi (Wanli) | M10 | Qin  Ca. 221-207 BC | Tibia | Female | 30-35 | 0.4 | -8.4 | 10.4 | 39.5 | 14.3 | 3.2 | Unknown | None |
| Liyi (Wanli) | M12 | Qin  Ca. 221-207 BC | Humerus | Female | 35-40 | 0.3 | -8.1 | 10.7 | 38.6 | 14.0 | 3.2 | Unknown | 1 bronze belt hook  3 common pottery |
| Liyi (Wanli) | M14 | Qin  Ca. 221-207 BC | Humerus | ? | 35 | 0.6 | -8.8 | 10.7 | 34.0 | 11.8 | 3.4 | Unknown | None |
| Liyi (Wanli) | M17 | Qin  Ca. 221-207 BC | Tibia | Female | Adult | 0.3 | -9.1 | 10.4 | 42.3 | 14.7 | 3.3 | Unknown | 2 checker pieces made of pottery |
| Liyi (Wanli) | M18 | Qin  Ca. 221-207 BC | Tibia | ? | ? | 0.4 | -8.9 | 10.3 | 34.6 | 12.1 | 3.3 | Unknown | 2 common pottery |
| Liyi (Wanli) | M19 | Qin  Ca. 221-207 BC | Tibia | Male | 35-39 | 0.3 | -8.3 | 10.4 | 40.9 | 14.8 | 3.2 | Unknown | None |
| Liyi (Wanli) | M20 | Qin  Ca. 221-207 BC | Rib | Female | 40-45 | 0.6 | -9.2 | 10.6 | 31.7 | 10.6 | 3.5 | Unknown | None |
| Liyi (Wanli) | M22 | Qin  Ca. 221-207 BC | Femur | Female | 50-55 | 0.7 | -8.4 | 9.9 | 42.4 | 15.5 | 3.2 | Unknown | None |
| Liyi (Wanli) | M23 | Qin  Ca. 221-207 BC | Tibia | Female | 40-45 | 0.7 | -7.8 | 10.2 | 42.5 | 15.5 | 3.2 | Unknown | 3 shells |
| Liyi (Wanli) | M24 | Qin  Ca. 221-207 BC | Humerus | ? | 30 | 0.5 | -8.1 | 10.5 | 40.2 | 14.4 | 3.3 | Unknown | None |
| Liyi (Wanli) | M26 | Qin  Ca. 221-207 BC | Rib | Male | 29-30 | 0.1 | -8.5 | 9.3 | 35.6 | 12.8 | 3.2 | Unknown | 2 jade objects  1 pottery jar |
| Liyi (Wanli) | M27 | Qin  Ca. 221-207 BC | Humerus | Male | 40-42 | 1.1 | -8.3 | 10.8 | 42.5 | 15.7 | 3.2 | Unknown | None |
| Liyi (Wanli) | M29 | Qin  Ca. 221-207 BC | Femur | Female | 45-50 | 0.6 | -8.1 | 9.9 | 41.9 | 15.3 | 3.2 | Unknown | 1 jade loop  4 common pottery |
| Liyi (Wanli) | M33 | Qin  Ca. 221-207 BC | Tibia | Female | 40-44 | 0.2 | -8.7 | 8.9 | 38.9 | 13.7 | 3.3 | Unknown | None |
| Liyi (Wanli) | M36 | Qin  Ca. 221-207 BC | Humerus | Female | Adult | 0.7 | -8.2 | 10.2 | 41.4 | 14.9 | 3.2 | Unknown | 3 common pottery |
| Liyi (Wanli) | M37 | Qin  Ca. 221-207 BC | Tibia | Male | 24-26 | 1.5 | -7.7 | 10.7 | 40.5 | 14.8 | 3.2 | Unknown | 4 common pottery |
| Liyi (Wanli) | M38 | Qin  Ca. 221-207 BC | Rib | ? | 35 | 0.8 | -8.2 | 9.8 | 42.5 | 15.4 | 3.2 | Unknown | 5 common pottery |
| Liyi (Wanli) | M42 | Qin  Ca. 221-207 BC | Tibia | Female | 35-40 | 0.3 | -7.5 | 10.9 | 31.4 | 11.0 | 3.3 | Unknown | 1 bronze belt hook  2 common pottery |
| Liyi (Wanli) | M45 | Qin  Ca. 221-207 BC | Rib | ? | ? | 0.4 | -8.3 | 10.6 | 42.5 | 15.5 | 3.2 | Unknown | 3 jade objects  1 bronze belt hook  3 common pottery |
| Liyi (Wanli) | M47 | Qin  Ca. 221-207 BC | Tibia | Male | 31-34 | 0.4 | -8.0 | 9.9 | 38.2 | 13.9 | 3.2 | Unknown | None |
| Liyi (Wanli) | M48 | Qin  Ca. 221-207 BC | Humerus | Male | 42 | 0.3 | -8.2 | 10.6 | 33.2 | 11.9 | 3.2 | Unknown | 1 bronze mirror  3 common pottery |
| Liyi (Wanli) | M51 | Qin  Ca. 221-207 BC | Rib | Male | 35-39 | 0.8 | -8.1 | 10.7 | 39.4 | 14.3 | 3.2 | Unknown | 4 common pottery |
| Liyi (Wanli) | M52 | Qin  Ca. 221-207 BC | Tibia | Female | 36-40 | 0.3 | -8.6 | 10.4 | 36.9 | 13.2 | 3.3 | Unknown | 2 common pottery |
| Liyi (Wanli) | M53 | Qin  Ca. 221-207 BC | Tibia | Male | 31-34 | 1.0 | -8.8 | 9.3 | 42.6 | 15.9 | 3.1 | Unknown | 1 bronze mirror  4 common pottery |
| Liyi (Wanli) | M54 | Qin  Ca. 221-207 BC | Femur | Male | 40-44 | 0.5 | -7.6 | 10.7 | 38.8 | 14.3 | 3.2 | Unknown | 9 common potter |
| Liyi (Wanli) | M55 | Qin  Ca. 221-207 BC | Tibia | Female | 35 | 0.5 | -7.9 | 9.6 | 42.3 | 15.0 | 3.3 | Unknown | 1 jade hair clasp  1 bone hair clasp |
| Liyi (Wanli) | M57 | Qin  Ca. 221-207 BC | Rib | Male | 40-44 | 1.1 | -7.9 | 11.0 | 43.0 | 15.7 | 3.2 | Unknown | 1 bronze mirror  4 common pottery |
| Liyi (Wanli) | M58 | Qin  Ca. 221-207 BC | Femur | Male | 30-35 | 0.5 | -9.0 | 10.3 | 42.4 | 15.0 | 3.3 | Unknown | 2 painted pottery  4 common pottery |
| Liyi (Wanli) | M59 | Qin  Ca. 221-207 BC | Humerus | Female | 50 | 0.5 | -8.8 | 9.8 | 42.4 | 15.5 | 3.2 | Unknown | 3 common pottery |
| Liyi (Wanli) | M62 | Qin  Ca. 221-207 BC | Tibia | Female | >60 | 3.2 | -8.4 | 11.0 | 42.8 | 15.8 | 3.2 | Unknown | None |
| Liyi (Wanli) | M72 | Qin  Ca. 221-207 BC | Humerus | ? | ? | 2.0 | -7.5 | 10.2 | 43.0 | 15.77 | 3.2 | Unknown | 1 bronze mirror  1 bronze loop  1 bronze spoon  2 iron knives  2 painted pottery  4 common pottery |
| Liyi (Wanli) | M74 | Qin  Ca. 221-207 BC | Tibia | Female | 35-39 | 2.3 | -8.7 | 10.5 | 42.2 | 15.56 | 3.2 | Unknown | 1 bronze mirror  1 common pottery |
| Liyi (Wanli) | M83 | Qin  Ca. 221-207 BC | Tibia | Female | 25 | 0.3 | -13.8 | 9.7 | 41.4 | 14.80 | 3.3 | Unknown | 1 bronze mirror  1 lead object  6 common pottery |
| Liyi (Wanli) | M84 | Qin  Ca. 221-207 BC | Humerus | Male | 40-45 | 0.7 | -9.0 | 10.8 | 49.1 | 16.69 | 3.4 | Unknown | 4 common pottery |
| Liyi (Wanli) | M86 | Qin  Ca. 221-207 BC | Femur | Female | 40-45 | 0.1 | -13.1 | 8.6 | 40.7 | 14.49 | 3.3 | Unknown | 5 common pottery |
| Liyi (Wanli) | M87 | Qin  Ca. 221-207 BC | Tibia | Male | 31-34 | 0.2 | -9.8 | 9.9 | 51.6 | 18.74 | 3.2 | Unknown | 1 iron belt hook |
| Liyi (Wanli) | M88 | Qin  Ca. 221-207 BC | Femur | Male | 31-34 | 0.3 | -8.9 | 9.8 | 43.9 | 15.56 | 3.3 | Unknown | 1 bronze belt hook  1 bronze loop  1 iron object  4 common pottery |
| Liyi (Wanli) | M91 | Qin  Ca. 221-207 BC | Tibia | Female | >60 | 0.2 | -7.3 | 10.1 | 51.7 | 18.45 | 3.3 | Unknown | 1 common pottery |
| Liyi (Wanli) | M93 | Qin  Ca. 221-207 BC | Humerus | Female | 45-50 | 0.4 | -8.9 | 10.2 | 49.4 | 17.53 | 3.3 | Unknown | None |
| Liyi (Wanli) | M94 | Qin  Ca. 221-207 BC | Humerus | Male | 31-34 | 0.2 | -7.9 | 10.5 | 48.8 | 17.25 | 3.3 | Unknown | 2 common pottery |
| Liyi (Wanli) | M97 | Qin  Ca. 221-207 BC | Humerus | Female | 40-44 | 0.1 | -7.0 | 12.0 | 50.5 | 17.79 | 3.3 | Unknown | 1 bronze belt hook |
| Liyi (Wanli) | M104 | Qin  Ca. 221-207 BC | Rib | ? | ? | 0.5 | -7.6 | 10.0 | 47.5 | 16.69 | 3.3 | Unknown | 2 common pottery |
| Liyi (Wanli) | M114 | Qin  Ca. 221-207 BC | Tibia | Female | 40-45 | 0.1 | -7.5 | 10.1 | 36.1 | 12.92 | 3.3 | Unknown | None |
| Liyi (Wanli) | M116 | Qin  Ca. 221-207 BC | Tibia | Male | >60 | 0.5 | -7.8 | 10.9 | 37.6 | 13.42 | 3.3 | Unknown | 1 bronze belt hook  1 iron belt hook  1 iron knife |
| Liyi (Wanli) | M25 | Qin  Ca. 221-207 BC | Tibia | Male | 22-23 | 0.2 | -11.3 | 10.0 | 48.2 | 17.13 | 3.3 | Unknown | 4 common pottery |
| **Samples below failed to produce collagen** | | | | | | | | | | | | | |
| Shanren | M89 | Qin  Ca. 221-207 BC | Rib | Male | 35-40 | -- | -- | -- | -- | -- | -- | Unknown | None |
| Shanren | M98 | Qin  Ca. 221-207 BC | Rib | Male | 34-35 | -- | -- | -- | -- | -- | -- | Impacted tooth | None |
| Shanren | M105 | Qin  Ca. 221-207 BC | Tibia | Male | 25 | -- | -- | -- | -- | -- | -- | Periapical abscess | None |
| Shanren | M106 | Qin  Ca. 221-207 BC | Rib | Male | 25 | -- | -- | -- | -- | -- | -- | Unknown | None |
| Shanren | M110 | Qin  Ca. 221-207 BC | Tibia | Male | 30-35 | -- | -- | -- | -- | -- | -- | Periodontitis,  Dental caries | None |
| Liyi (Xinfeng) | M44 | Qin  Ca. 221-207 BC | Humerus | Male | 60 | -- | -- | -- | -- | -- | -- | Unknown | 4 common pottery |
| Liyi (Xinfeng) | M174 | Qin  Ca. 221-207 BC | Humerus | Female | 41-50 | -- | -- | -- | -- | -- | -- | Unknown | 5 common pottery |
| Liyi (Xinfeng) | M84 | Qin  Ca. 221-207 BC | Femur | ? | ? | -- | -- | -- | -- | -- | -- | Unknown | 2 bronze vessels |
| Liyi (Xinfeng) | M85 | Qin  Ca. 221-207 BC | Humerus | ? | ? | -- | -- | -- | -- | -- | -- | Unknown | Unknown |
| Liyi (Xinfeng) | M91 | Qin  Ca. 221-207 BC | Tibia | ? | ? | -- | -- | -- | -- | -- | -- | Unknown | Unknown |
| Liyi (Xinfeng) | M93 | Qin  Ca. 221-207 BC | Femur | ? | ? | -- | -- | -- | -- | -- | -- | Unknown | Unknown |
| Liyi (Xinfeng) | M112 | Qin  Ca. 221-207 BC | Rib | ? | ? | -- | -- | -- | -- | -- | -- | Unknown | Unknown |
| Liyi (Xinfeng) | M151 | Qin  Ca. 221-207 BC | Tibia | ? | ? | -- | -- | -- | -- | -- | -- | Unknown | Unknown |
| Liyi (Xinfeng) | M178 | Qin  Ca. 221-207 BC | Femur | ? | ? | -- | -- | -- | -- | -- | -- | Unknown | Unknown |
| Liyi (Xinfeng) | M286 | Qin  Ca. 221-207 BC | Rib | ? | ? | -- | -- | -- | -- | -- | -- | Unknown | Unknown |
| Liyi (Xinfeng) | M304 | Qin  Ca. 221-207 BC | Tibia | ? | ? | -- | -- | -- | -- | -- | -- | Unknown | Unknown |
| Liyi (Xinfeng) | M307 | Qin  Ca. 221-207 BC | Tibia | ? | ? | -- | -- | -- | -- | -- | -- | Unknown | Unknown |
| Liyi (Xinfeng) | M310 | Qin  Ca. 221-207 BC | Rib | ? | ? | -- | -- | -- | -- | -- | -- | Unknown | Unknown |
| Liyi (Xinfeng) | M318 | Qin  Ca. 221-207 BC | Femur | ? | ? | -- | -- | -- | -- | -- | -- | Unknown | Unknown |
| Liyi (Xinfeng) | M328 | Qin  Ca. 221-207 BC | Femur | ? | ? | -- | -- | -- | -- | -- | -- | Unknown | Unknown |
| Liyi (Xinfeng) | M336 | Qin  Ca. 221-207 BC | Humerus | ? | ? | -- | -- | -- | -- | -- | -- | Unknown | Unknown |
| Liyi (Xinfeng) | M348 | Qin  Ca. 221-207 BC | Tibia | ? | ? | -- | -- | -- | -- | -- | -- | Unknown | Unknown |
| Liyi (Xinfeng) | M352 | Qin  Ca. 221-207 BC | Rib | ? | ? | -- | -- | -- | -- | -- | -- | Unknown | Unknown |
| Liyi (Xinfeng) | M353 | Qin  Ca. 221-207 BC | Tibia | ? | ? | -- | -- | -- | -- | -- | -- | Unknown | Unknown |
| Liyi (Xinfeng) | M440 | Qin  Ca. 221-207 BC | Rib | ? | ? | -- | -- | -- | -- | -- | -- | Unknown | Unknown |
| Liyi (Xinfeng) | M442 | Qin  Ca. 221-207 BC | Femur | ? | ? | -- | -- | -- | -- | -- | -- | Unknown | Unknown |
| Liyi (Xinfeng) | M461 | Qin  Ca. 221-207 BC | Rib | ? | ? | -- | -- | -- | -- | -- | -- | Unknown | Unknown |
| Liyi (Xinfeng) | M472 | Qin  Ca. 221-207 BC | Tibia | ? | ? | -- | -- | -- | -- | -- | -- | Unknown | Unknown |
| Liyi (Xinfeng) | M473 | Qin  Ca. 221-207 BC | Rib | ? | ? | -- | -- | -- | -- | -- | -- | Unknown | Unknown |
| Liyi (Xinfeng) | M479 | Qin  Ca. 221-207 BC | Tibia | ? | ? | -- | -- | -- | -- | -- | -- | Unknown | Unknown |
| Liyi (Xinfeng) | M480 | Qin  Ca. 221-207 BC | Rib | ? | ? | -- | -- | -- | -- | -- | -- | Unknown | Unknown |
| Liyi (Xinfeng) | M490 | Qin  Ca. 221-207 BC | Tibia | ? | ? | -- | -- | -- | -- | -- | -- | Unknown | Unknown |
| Liyi (Xinfeng) | M492 | Qin  Ca. 221-207 BC | Rib | Female | 50 | -- | -- | -- | -- | -- | -- | Unknown | 1 bronze vessel |
| Liyi (Xinfeng) | M493 | Qin  Ca. 221-207 BC | Femur | ? | ? | -- | -- | -- | -- | -- | -- | Unknown | Unknown |
| Liyi (Xinfeng) | M495 | Qin  Ca. 221-207 BC | Tibia | ? | ? | -- | -- | -- | -- | -- | -- | Unknown | Unknown |
| Liyi (Xinfeng) | M499 | Qin  Ca. 221-207 BC | Tibia | ? | ? | -- | -- | -- | -- | -- | -- | Unknown | Unknown |
| Liyi (Xinfeng) | M500 | Qin  Ca. 221-207 BC | Tibia | ? | ? | -- | -- | -- | -- | -- | -- | Unknown | Unknown |
| Liyi (Xinfeng) | M505 | Qin  Ca. 221-207 BC | Humerus | ? | ? | -- | -- | -- | -- | -- | -- | Unknown | Unknown |
| Liyi (Xinfeng) | M510 | Qin  Ca. 221-207 BC | Rib | ? | ? | -- | -- | -- | -- | -- | -- | Unknown | Unknown |
| Liyi (Xinfeng) | M514 | Qin  Ca. 221-207 BC | Tibia | ? | ? | -- | -- | -- | -- | -- | -- | Unknown | Unknown |
| Liyi (Xinfeng) | M516 | Qin  Ca. 221-207 BC | Tibia | ? | ? | -- | -- | -- | -- | -- | -- | Unknown | Unknown |
| Liyi (Xinfeng) | M517 | Qin  Ca. 221-207 BC | Rib | ? | ? | -- | -- | -- | -- | -- | -- | Unknown | Unknown |
| Liyi (Xinfeng) | M520 | Qin  Ca. 221-207 BC | Humerus | ? | ? | -- | -- | -- | -- | -- | -- | Unknown | Unknown |
| Liyi (Xinfeng) | M521 | Qin  Ca. 221-207 BC | Tibia | ? | ? | -- | -- | -- | -- | -- | -- | Unknown | Unknown |
| Liyi (Xinfeng) | M529 | Qin  Ca. 221-207 BC | Tibia | ? | ? | -- | -- | -- | -- | -- | -- | Unknown | Unknown |
| Liyi (Xinfeng) | M530 | Qin  Ca. 221-207 BC | Rib | ? | ? | -- | -- | -- | -- | -- | -- | Unknown | Unknown |
| Liyi (Xinfeng) | M533 | Qin  Ca. 221-207 BC | Rib | ? | ? | -- | -- | -- | -- | -- | -- | Unknown | Unknown |
| Liyi (Xinfeng) | M535 | Qin  Ca. 221-207 BC | Tibia | ? | ? | -- | -- | -- | -- | -- | -- | Unknown | Unknown |
| Liyi (Xinfeng) | M536 | Qin  Ca. 221-207 BC | Humerus | ? | ? | -- | -- | -- | -- | -- | -- | Unknown | Unknown |
| Liyi (Xinfeng) | M537 | Qin  Ca. 221-207 BC | Tibia | ? | ? | -- | -- | -- | -- | -- | -- | Unknown | Unknown |
| Liyi (Xinfeng) | M538 | Qin  Ca. 221-207 BC | Tibia | ? | ? | -- | -- | -- | -- | -- | -- | Unknown | Unknown |
| Liyi (Xinfeng) | M541 | Qin  Ca. 221-207 BC | Tibia | ? | ? | -- | -- | -- | -- | -- | -- | Unknown | Unknown |
| Liyi (Xinfeng) | M543 | Qin  Ca. 221-207 BC | Rib | ? | ? | -- | -- | -- | -- | -- | -- | Unknown | Unknown |
| Liyi (Xinfeng) | M544 | Qin  Ca. 221-207 BC | Femur | ? | ? | -- | -- | -- | -- | -- | -- | Unknown | Unknown |
| Liyi (Xinfeng) | M545 | Qin  Ca. 221-207 BC | Femur | ? | ? | -- | -- | -- | -- | -- | -- | Unknown | Unknown |
| Liyi (Xinfeng) | M547 | Qin  Ca. 221-207 BC | Rib | ? | ? | -- | -- | -- | -- | -- | -- | Unknown | Unknown |
| Liyi (Xinfeng) | M549 | Qin  Ca. 221-207 BC | Tibia | ? | ? | -- | -- | -- | -- | -- | -- | Unknown | Unknown |
| Liyi (Xinfeng) | M551 | Qin  Ca. 221-207 BC | Femur | ? | ? | -- | -- | -- | -- | -- | -- | Unknown | Unknown |
| Liyi (Xinfeng) | M557 | Qin  Ca. 221-207 BC | Femur | ? | ? | -- | -- | -- | -- | -- | -- | Unknown | Unknown |
| Liyi (Xinfeng) | M558 | Qin  Ca. 221-207 BC | Tibia | ? | ? | -- | -- | -- | -- | -- | -- | Unknown | Unknown |
| Liyi (Xinfeng) | M569 | Qin  Ca. 221-207 BC | Tibia | ? | ? | -- | -- | -- | -- | -- | -- | Unknown | Unknown |
| Liyi (Xinfeng) | M569 | Qin  Ca. 221-207 BC | Femur | ? | ? | -- | -- | -- | -- | -- | -- | Unknown | Unknown |
| Liyi (Xinfeng) | M573 | Qin  Ca. 221-207 BC | Femur | ? | ? | -- | -- | -- | -- | -- | -- | Unknown | Unknown |
| Liyi (Xinfeng) | M577 | Qin  Ca. 221-207 BC | Humerus | ? | ? | -- | -- | -- | -- | -- | -- | Unknown | Unknown |
| Liyi (Xinfeng) | M584 | Qin  Ca. 221-207 BC | Tibia | ? | ? | -- | -- | -- | -- | -- | -- | Unknown | Unknown |
| Liyi (Xinfeng) | M592 | Qin  Ca. 221-207 BC | Humerus | ? | ? | -- | -- | -- | -- | -- | -- | Unknown | Unknown |
| Liyi (Xinfeng) | M592 | Qin  Ca. 221-207 BC | Tibia | ? | ? | -- | -- | -- | -- | -- | -- | Unknown | Unknown |
| Liyi (Xinfeng) | M600 | Qin  Ca. 221-207 BC | Rib | ? | ? | -- | -- | -- | -- | -- | -- | Unknown | Unknown |
| Liyi (Xinfeng) | M603 | Qin  Ca. 221-207 BC | Tibia | ? | ? | -- | -- | -- | -- | -- | -- | Unknown | Unknown |
| Liyi (Xinfeng) | M634 | Qin  Ca. 221-207 BC | Femur | ? | ? | -- | -- | -- | -- | -- | -- | Unknown | Unknown |
| Liyi (Xinfeng) | M635 | Qin  Ca. 221-207 BC | Tibia | ? | ? | -- | -- | -- | -- | -- | -- | Unknown | Unknown |
| Liyi (Xinfeng) | M649 | Qin  Ca. 221-207 BC | Tibia | ? | ? | -- | -- | -- | -- | -- | -- | Unknown | Unknown |
| Liyi (Xinfeng) | M665 | Qin  Ca. 221-207 BC | Femur | ? | ? | -- | -- | -- | -- | -- | -- | Unknown | Unknown |
| Liyi (Xinfeng) | M668 | Qin  Ca. 221-207 BC | Tibia | ? | ? | -- | -- | -- | -- | -- | -- | Unknown | Unknown |
| Liyi (Xinfeng) | M710 | Qin  Ca. 221-207 BC | Tibia | ? | ? | -- | -- | -- | -- | -- | -- | Unknown | Unknown |
| Liyi (Xinfeng) | M706 | Qin  Ca. 221-207 BC | Humerus | ? | ? | -- | -- | -- | -- | -- | -- | Unknown | Unknown |
| Liyi (Wanli) | M43 | Qin  Ca. 221-207 BC | Tibia | Male | 24-26 | -- | -- | -- | -- | -- | -- | Unknown | 3 common potter |
| Liyi (Wanli) | M64 | Qin  Ca. 221-207 BC | Rib | Female | Adult | -- | -- | -- | -- | -- | -- | Unknown | 1 bronze mirror  2 shell  2 bead  1 lacquer ware  7 common pottery |
| Liyi (Wanli) | M89 | Qin  Ca. 221-207 BC | Tibia | Male | 40-44 | -- | -- | -- | -- | -- | -- | Unknown | None |
| Liyi (Wanli) | M90 | Qin  Ca. 221-207 BC | Rib | Female | 42 | -- | -- | -- | -- | -- | -- | Unknown | None |
| Liyi (Wanli) | M96 | Qin  Ca. 221-207 BC | Femur | ? | Adult | -- | -- | -- | -- | -- | -- | Unknown | 1 bronze belt hook |
| Liyi (Wanli) | M112 | Qin  Ca. 221-207 BC | Tibia | Female | 30 | -- | -- | -- | -- | -- | -- | Unknown | 1 bronze belt hook  2 common pottery |
